# Alteration in Light Spectra Causes Opposite Responses in Volatile Phenylpropanoids and Terpenoids Compared with Phenolic Acids in Sweet Basil (*Ocimum basilicum*) Leaves

**DOI:** 10.1021/acs.jafc.2c03309

**Published:** 2022-09-20

**Authors:** Minna Kivimäenpä, Adedayo Mofikoya, Ahmed M. Abd El-Raheem, Johanna Riikonen, Riitta Julkunen-Tiitto, Jarmo K. Holopainen

**Affiliations:** † Department of Environmental and Biological Sciences, University of Eastern Finland, P.O. Box 1627, 70211 Kuopio, Finland; ‡ Department of Economic Entomology and Agricultural Zoology, Menoufia University, Shebin El Kom 32514, Egypt; § Natural Resources Institute Finland, Juntintie 154, 77600 Suonenjoki, Finland; ∥ Department of Environmental and Biological Sciences, University of Eastern Finland, P.O. Box 111, 80101 Joensuu, Finland

**Keywords:** basil (*Ocimum basilicum* L.), light spectra, light-emitting diodes, secondary chemistry, phenolics, phenolic acids, terpenoids, leaf anatomy, glandular trichome, photosynthesis, pigment

## Abstract

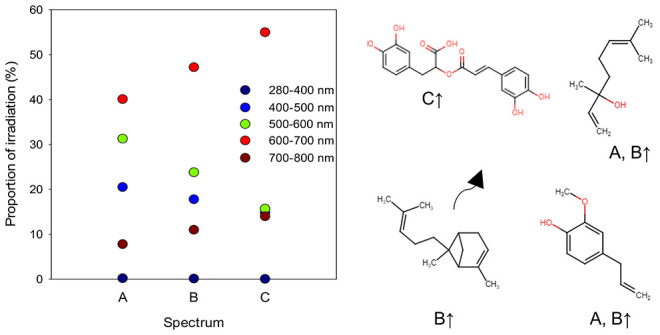

Basil (*Ocimum basilicum*, cv. Dolly) grew under three different light spectra (A, B, and C) created by light-emitting diode lamps. The proportions of UV-A, blue, and green-yellow wavelengths decreased linearly from A to C, and the proportions of red and far-red wavelengths increased from A to C. Photosynthetic photon flux density was 300 μmol m^–2^ s^–1^ in all spectra. The spectrum C plants had highest concentrations of phenolic acids (main compounds: rosmarinic acid and cichoric acid), lowest concentrations and emissions of phenylpropanoid eugenol and terpenoids (main compounds: linalool and 1,8-cineole), highest dry weight, and lowest water content. Conversely, spectra A and B caused higher terpenoid and eugenol concentrations and emissions and lower concentrations of phenolic acids. High density of peltate glandular trichomes explained high terpenoid and eugenol concentrations and emissions. Basil growth and secondary compounds affecting aroma and taste can be modified by altering light spectra; however, increasing terpenoids and phenylpropanoids decreases phenolic acids and growth and vice versa.

## Introduction

Sweet basil (*Ocimum basilicum*) is a widely used herb for culinary purposes and in traditional medicine.^1^ The distinctive aroma and fragrance of basil are due to secondary compounds such as phenolic acids (rosmarinic acid, cichoric acid, caffeic acid, and caftaric acid), volatile phenylpropanoids (eugenol and methylchavicol), and volatile terpenoids (including monoterpenes limonene, linalool, 1,8-cineole, and camphor and sesquiterpenes bergamotene and caryophyllene) but the composition varies between cultivars.^1−4^ Many secondary chemicals protect plants from herbivores, fungal and microbial diseases, as well as from abiotic stresses, by acting, e.g., as antioxidants and antibiotics,^5−7^ and the therapeutic influence of basil in the traditional medicine may be based on these properties.^1,4,8^ Volatile phenylpropanoids and terpenoids of basil leaves are stored in the peltate glandular trichomes with four secretory cells.^9^ Basil also has capitate glandular trichomes with one or two secretory cells^10^ that were reported to contain straight-chain hydrocarbons and small-chain alcohols.^9^ Phenolic acids are synthetized both in the peltate glandular trichomes on the leaf surface and within the leaf tissues.^11^

In countries with short growing season, such as Finland, basil is mainly grown in greenhouses under partly artificial light. LED (light-emitting diode) technology has been adopted in greenhouses as an additional light source, but also as a sole light source under closed growing conditions, the so-called plant factories.^12^ LED light spectra can be modified to give only selected wavelengths for the plants.^13^ The first plant LEDs consisted of blue (400–500 nm) and red (600–700 nm) wavelengths based on their well-known influence on photosynthesis and growth. Current “white light” plant LEDs mix differently colored LEDs that combine various wavelengths, including green-yellow (500–600 nm) and far-red (700–800 nm) but have sufficient proportions of blue and red for photosynthesis. The appearance of the vegetables is more pleasing for the human eye and the condition of the vegetables is easier to observe under such spectra.^14^ Moreover, plants grow and photosynthesize better when blue and red wavelengths are supplemented with green, yellow, or far-red wavelengths.^15,16^

Various wavelengths of (LED) light are absorbed and sensed by various pigments and photoreceptors. Photosynthetic pigments, chlorophyll a and b, absorb blue and red wavelengths, and carotenoids absorb blue-green wavelengths.^17^ Various photoreceptors, for example, phototropin and cryptochrome for UV-A (315–400 nm) and blue and phytochrome for red and far-red wavelengths,^17^ control the physiological, morphological, growth, reproductive, and defense responses of plants, although responses to various wavelengths or their ratios depend on plant species, genotypes, and developmental phases of the plant.^17−19^ Blue wavelengths, in general, make plants shorter and leaves thicker by increasing palisade tissue thickness and epidermal cell size, increase content of carotenoids and stomatal density and phenolics (particularly flavonoid and anthocyanin) synthesis.^17,18^ Influence of red wavelengths is largely affected by the ratio of red and far-red wavelengths. The low red:far-red ratio makes plants more elongated and leaves larger but thinner (reduced mesophyll), decreases stomatal density, chlorophyll content, and photosynthesis.^19^

LED spectra can be used, e.g., to increase biomass and change the morphology of basil^20^ and consequently affect customer preferences. LEDs with specific spectra can also be used to modify chemical composition of plants during growth and post-harvest, and thus, potentially alter taste and aroma, as well as health-promoting or therapeutic benefits of food.^13,20,21^ In basil, LED lights have been shown to increase total phenolic concentrations of leaves compared to fluorescent lamps.^22^ Blue light, in general, increases concentrations of phenolics in many agricultural and ornamental species,^21^ but the blue-light response was not observed in basil and red light was suggested to have an important role in regulating phenolics accumulation in basil.^23^ LED lamps with a lower red:far-red ratio resulted in higher concentrations of rosmarinic acid in basil.^24^ Basil grown under monochromatic red light were reported to have lower myrcene, 1,8-cineol, γ-terpinene, linalool, and total content of essential oils than basil grown under monochromatic blue light or white light.^25^ The influence of the red:blue ratio on basil volatiles was studied and reduction in relative content of several volatile compounds, including the major compound linalool, in the lowest red:blue ratio of 0.5 (corresponding to the blue:red ratio of 2) was reported.^26^ Abundance of several volatile terpenoids and phenylpropanoids was higher in basil grown under narrow-band spectra LEDs than in a glass greenhouse.^15^ The study also showed that adding yellow or green wavelengths to blue and red wavelengths increased several monoterpenes (e.g., α-pinene, sabinene, 1,8-cineole, and α-terpineol), sesquiterpenes (e.g., α-bergamotene, α-humulene, and β-caryophyllene), and phenylpropanoids (e.g., eugenol and estragole) compared with blue and red wavelength spectra and adding the far-red wavelength increased sesquiterpenes.^15^ Although VOCs (volatile organic compounds) were collected from detached basil shoots,^15^ which is relevant from a consumer point of view, mechanical damage from the detachment causes fast release of green leaf volatiles (GLVs) due to membrane damage^27^ and may have masked some of the light spectra effects.

Effects of light spectra on both phenolics and terpenoids at a compound level simultaneously have seldom been studied. However, separate studies suggest that blue light increasing terpenoids in basil leaves seems not to have the same effect in phenolics.^23,25,26^ Increased flavonoids and acetophenones but reduced terpenoids and alkaloids were reported in Norway spruce (*Picea abies*) needles exposed to blue-light increment, and a shift in secondary metabolism processes was suggested.^28^ Additionally, molecular biological analysis of glandular trichomes of basil lines showed low expression of the enzymes of the shikimate/phenylpropanoid pathway when the expression for terpenoids was high.^29^ There is a need to study effects of light spectra on nonvolatile and volatile phenolic and terpenoid compounds simultaneously, because metabolic shifts or other changes in compound composition may affect the taste and aroma of basil.

The main aim was to study how commercially used plant LED lamps with the same light intensity but with linearly increasing or decreasing ratios and proportions of blue, yellow-green, red, and far-red wavelengths in the continuous spectra affect concentrations of nonvolatile phenolics and volatile phenylpropanoids and terpenoids in sweet basil leaves. Our main hypothesis was that under spectral conditions, where terpenoids increase, the phenolics decrease, and vice versa. In addition, we measured basil growth, biomass, and water content that are commercially important plant features affecting customer preferences. Moreover, we studied leaf physiology (gas exchange and photosynthetic pigments) and anatomy (tissue thicknesses, stomata, and trichome densities) as these are affected by light spectra and interconnected with leaf water relations and carbon allocation for growth and for accumulation of secondary metabolites.

## Materials and Methods

### Plant Material and Growing Conditions

Three seeds of basil (*Ocimum basilicum* cv. Dolly) (Enza Zaden, The Netherlands) were sown in 1 L pots filled with a mixture of peat (Kekkilä Puutarhaturve, Finland), soil (Kekkilä Puutarhamulta, Finland), sand (0.5–1.2 mm, Weber Saint-Gobain, Finland) in 3:1:1 (v:v:v). In total, 93 pots were sown. The pots were distributed to six boxes (40 × 65 cm), 15–16 pots to one box. The boxes were distributed to three closed computer-controlled growing chambers (Fitotron, Weiss Technik, Germany); thus, there were three chambers each with two boxes and in total 31 basil pots. The boxes were used to make rotation of plants within and between chambers (see below) easier and reduce risk of handling damage. Pots were given a running number that was later used for randomizing plants for various measurements and analyses.

**Figure 1:**
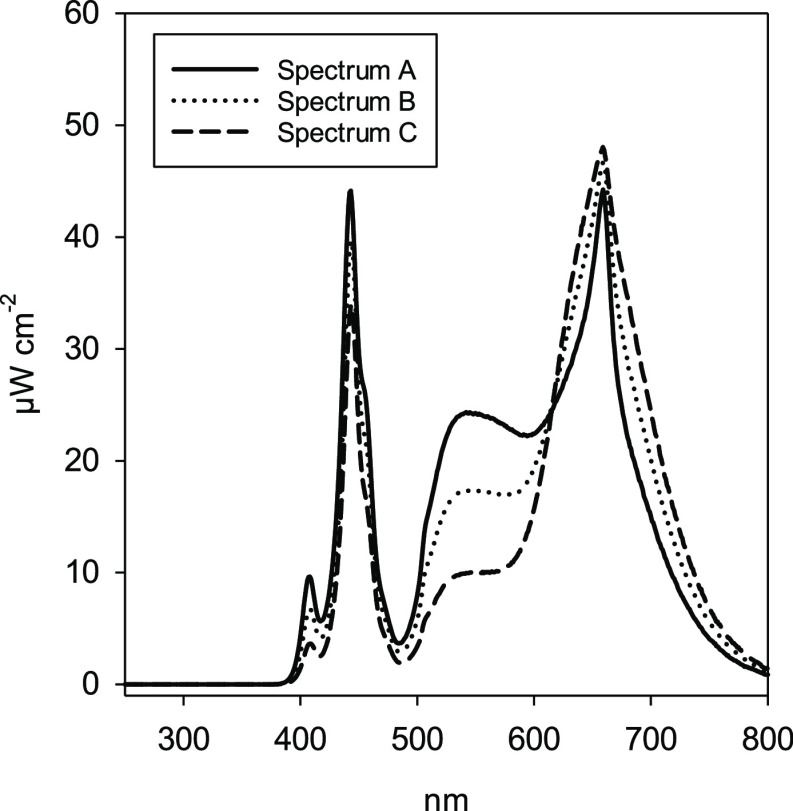
Spectral distribution (6 am–6 pm) of light under three light treatment spectra measured at 1 m distance of the LED bars.

Each chamber was lighted by six LED bars with continuous spectra (B100 series, Valoya Oy, Helsinki, Finland), three bars with spectra G2 and three bars with spectra NS1, positioned alternately on the ceiling. G2 has high proportion of red (70%) and far-red wavelengths (21%), the red:far-red ratio is 3.0 and the proportion of blue (7%) and green (2%) wavelengths low. Compared with G2, NS1 has a lower proportion of red (37%) and far-red (4%), a higher red:far-red ratio (10.0), a proportion of blue (22%) and green (36%) wavelengths, and it includes 1% UV-A (source of spectra for G2 and NS1: https://www.valoya.com/wp-content/uploads/2022/01/EN_Product-Brochure_2021.2.pdf). Daily light rhythm simulating summer months in Finland and three different spectral treatments named spectra A, B, and C were programmed by increasing or decreasing the power of the lamps (Figure 1, Table 1). Lights were switched off between 11 pm and 1 am. At 1 am, lamps were switched on and the power of the lamps in all chambers was 10%. After this, the power was increased linearly until 6 am as follows: in spectrum A, the power of G2 increased to 30% and that of NS1 to 70%, in spectrum B, the power of both G2 and NS1 were increased to 50%, and in spectrum C, the power of G2 was increased to 70% and that of NS1 to 30% (Table 1). The light level was kept at the constant level (photosynthetic photon flux density, PPFD at a wavelength range 400–700 nm, target 300 μmol m^–2^ s^–1^ at the top canopy level) between 6 am and 6 pm in all spectral treatments. After this, the light level linearly decreased until 11 pm so that power of all the lamps was 10% at the end. PPFD was 60 μmol m^–2^ s^–1^ when light went on or off and then the spectra was the same as in spectrum B in all light treatments. PPFD was measured and monitored using a photometer (Delta OHM, model HD 2102.2, Padova, Italy). The spectra were determined under constant light conditions (6 am–6 pm) using a high accuracy UV–visible spectroradiometer (Optronic Laboratories, USA) at the top canopy level, i.e., 1 m from the lamps. Wavelength ratios were estimated from spectral irradiance data,^30^ using R^31^ and the packages “photobiology” and “photobiologyPlants”.^32^ The light treatments A, B, and C formed a gradient of decreasing blue and green-yellow wavelengths and increasing red wavelengths from A to C (Table 1). Thus, treatment A had the highest blue:red ratio and C the lowest. At the same time, the red:far-red ratio decreased and the blue:green ratio increased from A to C (Table 1). A small fraction of UV was from the UV-A range (315–400 nm).

**Table 1:** Proportion (%) of Irradiation at Different Wavelength Ranges from Total Irradiation (W m^–2^) between 280 and 800 nm, and Ratios of Different Wavelength Ranges in the Three Light Treatments (6 am–6 pm)^a^^e^

	spectrum A	spectrum B	spectrum C
	G2:NS1 30:70	G2:NS1 50:50	G2:NS1 70:30
280–400 UV	0.20	0.13	0.06
400–500 blue	20.5	17.8	14.8
500–600 green and yellow	31.3	23.8	15.7
600–700 red	40.1	47.2	55.0
700–800 far-red	7.8	11.0	14.0
blue:red^b^	0.43	0.33	0.24
blue:green^c^	0.68	0.82	1.14
red:far-red^d^	5.21	4.16	3.52
Pfr:Ptot	0.77	0.77	0.76

a G2 and NS1 and ratio values refer to two different LED light bar types and their power ratios.

b 420–490 nm:620–680 nm.

c 420–490 nm:500–570 nm.

d 655–665 nm:725–735 nm.^30^

e Original spectra of G2 and NS1 available in https://www.valoya.com/wp-content/uploads/2022/01/EN_Product-Brochure_2021.2.pdf.

In all light spectra treatments, chamber target RH was 50% during the day (9 am–5 pm), increased to 80% until 11 pm, and started to decrease to 50% again at 3 am. The daytime target temperature was 23 °C between 9 am and 6 pm, after which temperature gradually decreased to 18 °C until 1 am and started to increase again after 4 am. Temperature deviation was 0.5 °C and RH deviation 3–5%-unit. Basil was grown under these chamber conditions for 2 months.

The position of the boxes within a chamber was changed every day. Moreover, the treatments between the chambers were changed weekly and plants were transferred to new chambers accordingly. The distance of the lamps in relation to the top of growing plants was adjusted to keep the PPFD at the target level throughout the experiment. The pots were watered every day and fertilized once a week after germination (cotyledon formed) with 0.1% solution of Taimi-Superex (N:P:K 19:4:20, Kekkilä, Finland). On the 16th day, when the first true leaves were formed, two plants from the three seeds sown in each pot were taken out and one average-looking plant was left to grow.

Thirty-one plants per spectra were distributed for various measurements as follows: 10 to 11 plants for growth, morphology, gas exchange, leaf anatomy, pigment, and phenolic concentrations; 10 plants for terpene concentration analyses; and 10 plants for analysis of VOCs, total biomass (fresh weight, FW, and dry weight, DW), DW %, and water content.

### Growth and Morphology Measurements

On day 56, 11 plants from each treatment were measured for plant height, average internode distance between the second and fifth leaves from the base, leaf and petiole lengths and leaf width from the second and third leaves from the base. Total biomass (FW and DW) and DW % and water content were determined from 10 plants used in VOC collection (see below).

### Leaf Gas Exchange

Net photosynthesis (Pn) and stomatal conductance (gs) were measured from 10 basil plants per treatment on day 55 from the beginning of the experiment using a portable photosynthesis system (LI-6400XT, Li-COR Inc., Lincoln, Nebraska, USA). Leaf temperature was set to 23 °C, stomatal ratio 0.5, and CO_2_ level 400 ppb. A Li-COR 6400-02B LED source and a saturating PAR (photosynthetically active radiation) level 500 μmol m^–2^ s^–1^ were used. Two youngest fully developed leaves from the uppermost part of the plant were measured and an average of two leaves was calculated. The ratio of Pn:gs was calculated to estimate water use efficiency.

### Leaf Anatomy

Two leaves from 10 plants per treatment were sampled for microscopy analyses on day 55. The leaves were of the same age as used for the gas exchange analyses.

#### Light Microscopy (LM)

Leaf segment of 1 × 1.5 mm next to the midrib was cut with a razor blade and put in a cold (+4 °C) prefixative containing 2.5% glutaraldehyde on 0.1 M cacodylate buffer (pH 7.2) overnight. The next day, samples were processed with a Lynx Microscopy Tissue Processor (Reichert-Jung Optische Verke AG, Wien, Austria) as follows: 0.1 M cacodylate buffer 2 × 15 min (+4 °C), 1% osmiumtetroxide in 0.1 M cacodylate buffer for 4 h (+4 °C), 0.1 M cacodylate buffer 3 × 10 min (+4 °C), increasing ethanol series (30, 50, 70, 94, and 100%) each 2 × 15 min (+4 °C), propylene oxide 2 × 15 min (+20 °C), propylene oxide:epon (Ladd LX112) 3:1 for 1 h (+20 °C), propylene oxide:epon 1:1 for 1 h (+20 °C), propylene oxide:epon 1:3 for 2 h (+20 °C), and pure epon overnight (+20 °C). Samples were embedded to epon in silicon molds and polymerized at +60 °C for 3 days. Sectioning and staining first in toluidine blue and then in *p*-phenylene diamine for LM were done as described earlier.^33^ The sections were photographed by a light microscope (Carl Zeiss Axio Imager M2, camera Axiocam MRc, Jena, Germany) using a 20× objective. Leaf thickness, palisade, spongy, upper epidermis, and lower epidermis thicknesses were measured from three locations per sample, and the sample and plant averages were calculated. For epidermises, the visibly thickest, narrowest, and most average-looking cells were measured. The proportion of epidermis cells filled with phenolic compounds (>50% vacuole area) was determined. The proportion of intercellular space from palisade and spongy tissues were also determined. Tools of ImageJ 1.47v were used in image analyses for LM and scanning electron microscopy (SEM).

#### Scanning Electron Microscopy

Rest of the leaves sampled for LM were air-dried, and two rectangular segments (ca. 3 × 3 mm) were cut next to the midrib (another side of the leaf than that used for LM) and placed on a self-adhesive copper tape on aluminum stubs, one with adaxial (upper) side and the other abaxial (lower) side upward. Samples were sputtered with ca. 50 nm layer of gold (Automatic Sputter Coater B7341, Agar Scientific Ltd., Stansted, UK). To evaluate the impact of leaf shrinking, samples of a few fresh leaves were prepared using chemical fixation for SEM (Supplementary method description). Samples were studied by high-resolution scanning electron microscopy **(**HR-SEM; Carl Zeiss, Sigma HD|VP, Oberkochen, Germany) using an SE2 detector and electron high tension 5.0 kV.

**Figure 2:**
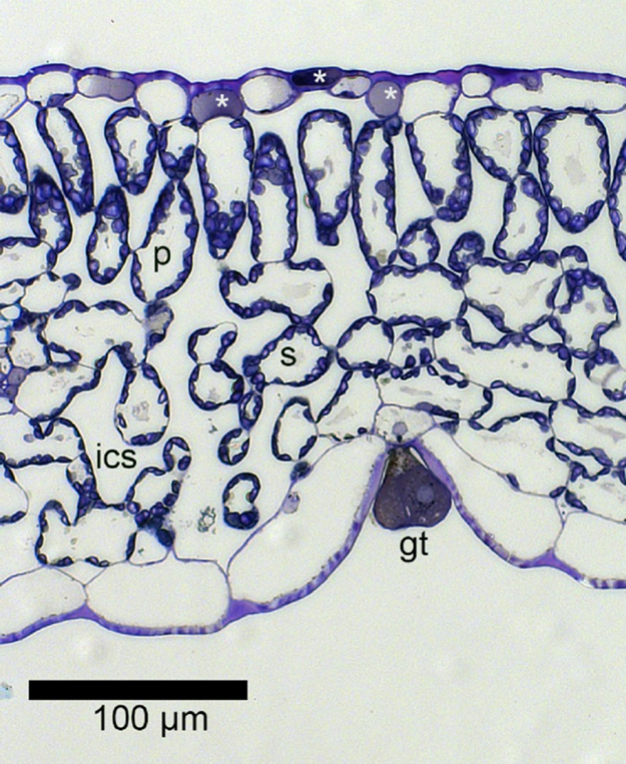
Cross-section of a basil leaf grown under spectrum C. White asterisks indicate upper epidermis cells with accumulation of phenolic compounds. gt = small glandular trichome sunken in the lower epidermis, p = palisade cell, s = spongy cell, and ics = intercellular space.

**Figure 3:**
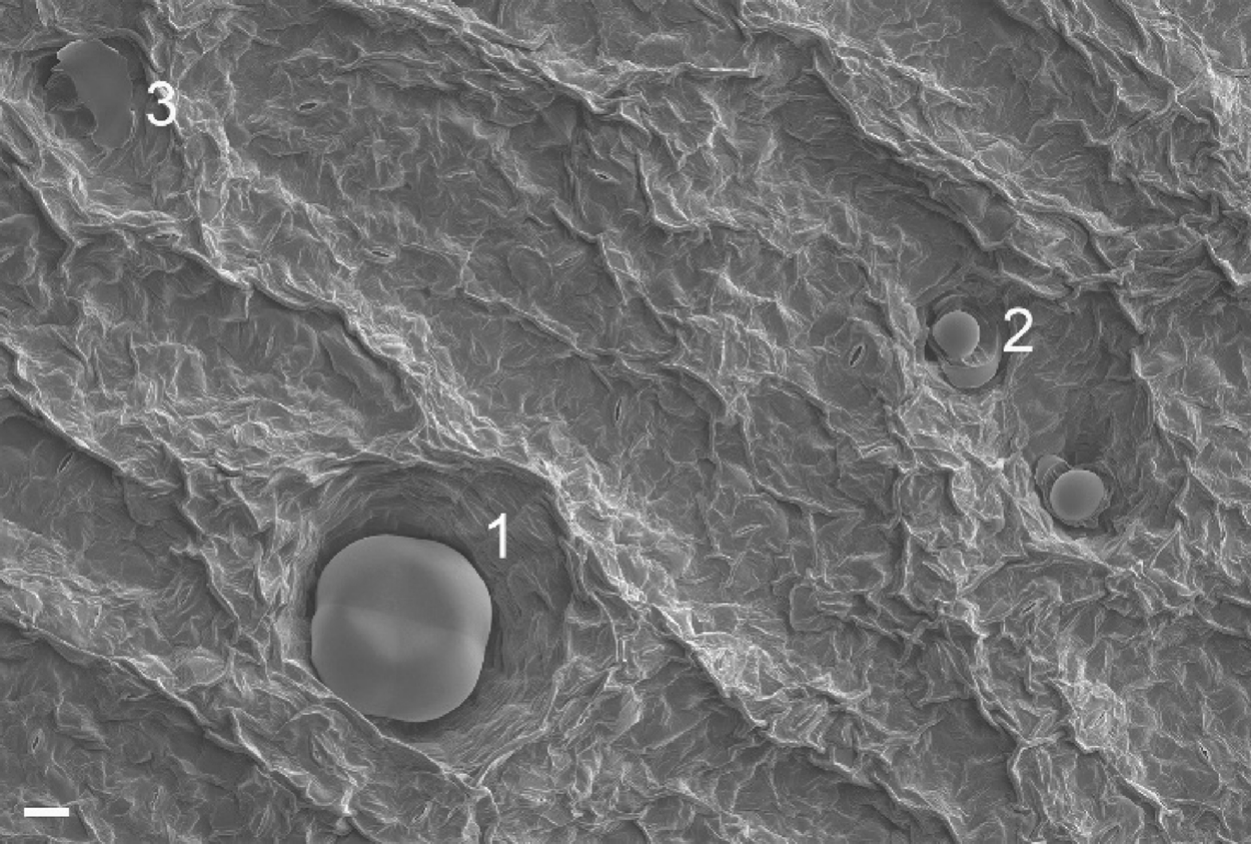
Glandular trichomes on a basil leaf surface: (1) large, intact peltate glandular trichome, (2) capitate glandular trichome or smaller developing peltate trichome, and (3) remnants of deteriorated trichome. Bar 20 μm. Air-dried sample.

Stomatal density (number per area) on both leaf surfaces was measured from three photographs taken at 1000× magnification, each showing a 56,500 μm^2^ leaf area. Glandular trichomes were of two size categories, 20–30 μm in diameter and 80–90 μm in diameter, often half-sunken in the epidermis (Figures 2 and 3). The larger glandular trichomes were regarded as fully developed peltate glandular trichomes due to the fourfold symmetry. The smaller glandular trichomes were either developing peltate glandular trichomes or capitate glandular trichomes. Some of the trichomes had lost their glandular form and their remnants were seen in and around the dints (Figure 2). The density of total glandular trichomes, and density of intact-looking larger peltate glandular trichomes were calculated from photographs taken at 150× magnification, each showing a 2.6 mm^2^ leaf area.

### Pigment and Phenolics Analyses

Samples for pigment analyses were collected from 11 plants per treatment on day 56. Three leaf disks were drilled from interveinal areas in the middle of the fully developed leaves from three uppermost whorls. Leaf disks (total FW ca. 80 mg) were frozen in liquid nitrogen, stored at −80 °C and analyzed for concentrations of chlorophyll a, chlorophyll b, and total carotenoids (carotenes and xanthophylls). The rest of the three leaves for each species were freeze-dried (Freeze dryer Alpha 1–2; Christ, Osterode am Harz, Germany) and used for anthocyanin and leaf phenolics analyses. Chlorophyll a, chlorophyll b, and total carotenoids from the leaf disks were extracted in 95% ethanol in the darkn at +4 °C for 24 h and measured spectrophotometrically^34^ and the concentration was calculated per DW. Anthocyanin samples were extracted in acidified methanol at +4 °C for 48 h and the concentration was determined spectrophotometrically.^35^ Phenolics were extracted in cold methanol from dry leaf samples and analyzed by HPLC and identified by QTOFMS.^36^

### Terpene Concentration Analysis

Two leaves from the second and third whorl from the top were detached on day 57 and collected to a cryotube containing liquid nitrogen and stored at −80 °C. Samples were collected from 10 plants, but some of the cryotubes had broken at −80 °C, thus *n* = 7 for spectrum A, *n* = 6 for spectrum B, and *n* = 8 for spectrum C. Samples were cut into small pieces in liquid nitrogen and 200 mg of the leaf sample was extracted in 2 mL of *n*-hexane containing 73.4 μg of 1-chloro octane as the internal standard at room temperature for 2 h. The extract was filtered and washed twice with 2 mL *n*-hexane. The extracts were analyzed using an Agilent 6890N (China) gas chromatograph equipped with a mass selective detector (type 5973 inert). Separations were carried out on a 30 m HP-5 ms 19091S-433 (i.d. 0.25 mm; film thickness 0.25 μm, Agilent, USA) column. Helium was used as the carrier gas, and linear velocity was about 36 cm s^–1^. The splitless (purge time off 0.45 min) sampling technique was used and 1 μL of sample was injected. The column temperature was programmed from 50 to 120 °C at 5 °C min^–1^, then to 210 °C at 15 °C min^–1^, then 280 °C at 30 °C min^–1^, and held for 7 min. Mass numbers from *m*/*z* 33 to 350 were recorded. Compounds were identified with help of 20 commercial standards, Wiley and NIST databases, and quantified with help of standard compounds using MSD ChemStation software. Quantification was based on total ion counts (TIC). The detection limit was ca. 0.1 μg for monoterpenes, 0.4 μg for sesquiterpenes, and 1 μg for (*E*)-2-hexenal. Compounds for which standards were not available, were quantified using α-pinene (nonoxygenated monoterpenes), 1,8-cineole (oxygenated monoterpenes), or α-copaene (sesquiterpenes) as references. Concentrations were calculated per DW.

### VOC Collections

VOC emission rates were measured from 10 intact plants per treatment on day 56 from the beginning of the experiment. Measurements were conducted in the growing chambers using custom-made collection systems.^37^ Plants were enclosed into disposable, precleaned (at +120 °C for 1 h) polyethylene terephthalate (PET) bags (25 × 55 cm, Look, Finland). The bag end was folded to provide a cushion for the stem and the bag was tied carefully around the stem with a shutter avoiding stem damage. Filtered and scrubbed air (500 mL min^–1^ for 10 min) was led to the bag via Teflon tubing through a hole cut to the other upper corner of the bag and tied with a shutter. A 6 L air sample (200 mL min^–1^ for 30 min) from the headspace was pulled into a steel tube filled with 250 mg of Tenax TA 60/80 and Carbopack B 60/80 (1:1 w:w, Markes International, UK) through a hole cut at the other corner of the bag. Air temperature inside the bags was monitored by wireless loggers (Hygrochron DS1923-f5 iButton, Maxim Integrated Products, San Jose, CA, USA). Bag enclosure increased air temperature by 1 °C compared with air temperature in the chambers. Blank samples were collected from empty bags. After VOC collection, plant parts inside the bag were cut, weighed fresh, dried at +60 °C for 72 h, and weighed again for calculation of VOC emission rates per DW. The rest of the plant was also weighed fresh and dry to calculate the total plant biomass. The samples were run with GC–MS after thermodesorption as earlier described^38^ and the detection limits were ca. 0.5 ng for monoterpenes, 2 ng for sesquiterpenes, and 4 ng for GLVs. Compound identification and quantification followed the same principles as of terpene samples. Emission rates per hour were calculated using formula 1
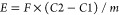
1where *E* is the emission rate (ng h^–1^ g^–1^ DW), *F* is the flow rate to the bag (L min^–1^), *C*2 is the concentration of the outgoing air (ng L^–1^), *C*1 is the concentration of the incoming air (ng L^–1^), *m* is the plant dry weight (g). *C*1 was considered to be 0 because the incoming air was filtered, and the quantities of VOCs determined from the empty collection bags were subtracted from the plant emission results.

### Statistics

Plant averages were calculated, and individual plant was used as a replicate in statistical analyses conducted by IBM SPSS 25. Data were examined for normality and homogeneity of the variances. Effects of light spectra were studied by one-way ANOVA with Tukey’s test for pairwise comparisons. The Welch test and Dunnett’s T3 test for pairwise comparisons were used if variances were not equal. The Kruskal–Wallis test with the Bonferroni test for pairwise comparisons was used if normality assumption was violated.

## Results

### Germination, Morphology, and Growth

All the basil seeds (of the seeds that eventually germinated) had germinated on the 12th day from sowing and light treatment did not affect the germination percentage (data not shown). On day 56, internodes were the longest and total plant biomass as dry weight and DW % were highest, and thus water content was the lowest, in the plants grown under spectrum C, and the differences were significant when compared with spectrum B plants (Table 2). Along with lowest DW and DW % (highest water content), leaves from spectrum B were the smallest (Table 2). Total DW was 47% higher in spectrum C than that in B and 17% higher in C than that in A. The petiole length or the plant height was not significantly affected (Table 2).

**Table 2:** Average (s.e.) (*n* = 11) Values for Growth Parameters of Basil Grown under Three Different LED Spectra on Day 56^a^

	spectrum A	spectrum B	spectrum C	*P*
height (cm)	40.3 (1.1)	39.4 (0.5)	42.2 (1.1)	0.115
aver. internode (cm)	9.6 (0.2)ab	9.5 (0.2)a	10.3 (0.3)b	**0.033**
aver petiole length (cm)	3.6 (0.1)	3.5 (0.1)	3.4 (0.1)	0.116
leaf width (cm)	11.9 (0.2)a	11.1 (0.2)b	11.6 (0.2)ab	**0.026**
leaf length (cm)	15.2 (0.3)a	14.2 (0.2)b	14.8 (0.3)ab	**0.026**
total FW^b^ (g)	39.9 (1.5)	36.1 (2.6)	42.7 (2.6)	0.143
total DW^b^ (g)	4.5 (0.2)ab	3.6 (0.4)a	5.3 (0.4)b	**0.011**
DW %	11.3 (0.3)ab	9.8 (0.6)a	12.2 (0.4)b	**0.003**

a *P*-values from ANOVA, *P* < 0.05 **emboldened**. Different letters indicate statistical difference (*P* < 0.05) between the treatments.

b FW = fresh weight, DW = dry weight. See Figure 1 and Table 1 for details of spectra.

### Gas Exchange and Pigments

Net photosynthesis (Pn) or stomatal conductance (gs) or their ratio, describing water use efficiency, was not significantly affected by the treatments (Table 3). Chlorophyll a and carotenoid concentrations were highest in spectrum B and lowest in C and the differences between all spectra were significant (Table 3). The ratio of chlorophyll a to chlorophyll b was higher in spectrum B than that in spectrum A. Anthocyanin concentrations tended to be the lowest in spectrum A.

**Table 3:** Average (s.e.) (*n* = 10–11) Net Photosynthesis (Pn), Stomatal Conductance (g_s_), Pn:g_s_ and Concentrations of Chlorophyll a, Chlorophyll b, Total Carotenoids, and Total Anthocyanins in Basil Leaves Grown under Three Different Light Spectra^a^

	spectrum A	spectrum B	spectrum C	*P*
Pn (μmol CO_2_ m^–2^ s^–1^)	4.5 (0.6)	3.7 (0.6)	3.2 (0.4)	0.306^b^
g_s_ (mol H_2_O m^–2^ s^–1^)	0.06 (0.01)	0.05 (0.01)	0.04 (0.01)	0.404^b^
Pn:g_s_	77.2 (4.1)	81.0 (4.3)	79.5 (5.6)	0.851^b^
Chl a (mg g^–1^ DW)	10.7 (0.2)a	12.1 (0.3)b	9.3 (0.3)c	**<0.001**^c^
Chl b (mg g^–1^ DW)	2.3 (0.1)	2.3 (0.1)	2.1 (0.2)	0.398^c^
Chl a:Chl b	4.7 (0.1)a	5.3 (0.2)b	4.7 (0.3)ab	0.050^c^
carotenoids (mg g^–1^ DW)	3.1 (0.1)a	3.6 (0.1)b	2.7 (0.1)c	**<0.001**^c^
anthocyanins (mg g^–1^ DW)	0.018 (0.003)	0.030 (0.004)	0.032 (0.005)	0.076^b^

a Different letters indicate statistical difference (*P* < 0.05) between the treatments.

b ANOVA.

c Welch test, *P* < 0.05 **emboldened**. See Figure 1 and Table 1 for details of spectra.

### Leaf Anatomy

Light spectra did not affect thicknesses of leaves or leaf tissues, the proportion of intercellular space, or stomatal or total trichome density in basil leaves (Table 4, Table 5). However, the density of large, intact-looking trichomes (Figure 3) on the lower leaf side was significantly lower in spectrum C than that in B (Table 5). Phenolics accumulation in the epidermis cells (Figure 2) tended to be higher in spectra C and A than that in spectrum B (Table 4). SEM samples prepared by drying had ca. 15% higher stomatal density and 25% higher trichome density than samples prepared by chemical fixation, because of cell shrinkage (data not shown). However, trichomes were better preserved in samples prepared by drying (data not shown).

**Table 4:** Average Values (s.e.) (*n* = 10) for Thicknesses of Leaves, Upper and Lower Epidermis and Palisade and Spongy Parenchyma, Proportion of Epidermis Cells with Phenolic Compounds, and Proportion of Intercellular Space (ics) of Mesophyll in Basil Leaves Grown under Three Different LED Spectra

	spectrum A	spectrum B	spectrum C	*P*
thickness (μm)				
leaf	260 (7)	266 (10)	253 (8)	0.546^a^
upper epidermis	23 (1)	23 (1)	23 (1)	0.748^a^
lower epidermis	24 (1)	26 (1)	25 (1)	0.085^a^
palisade	85 (3)	89 (5)	83 (4)	0.590^a^
spongy	133 (5)	130 (7)	125 (4)	0.561^a^
palisade:spongy	0.65 (0.03)	0.71 (0.05)	0.68 (0.03)	0.639^a^
proportion (%)				
phenolics upper epidermis	13 (4)	8 (3)	16 (4)	0.082^a^
phenolics lower epidermis	4 (1)	3 (2)	5 (1)	0.062^b^
ics palisade	29.9 (1.9)	29.3 (3.3)	25.5 (1.7)	0.376^a^
ics spongy	42.0 (1.6)	43.3 (1.4)	46.8 (2.5)	0.199^a^

a ANOVA.

b Kruskal–Wallis test. See Figure 1 and Table 1 for details of spectra.

**Table 5:** Average Values (s.e.) (*n* = 10) for Densities of Stomata and Trichomes on the Upper and Lower Sides of Basil Leaves Grown under Three Different LED Spectra^a^

	spectrum A	spectrum B	spectrum C	*P*
stomatal density (number per mm^2^)				
upper	171 (12)	190 (11)	205 (15)	0.177
lower	215 (10)	207 (11)	225 (15)	0.597
total trichome density (number per mm^2^)				
upper	7.9 (0.8)	6.7 (0.6)	7.3 (0.9)	0.608
lower	12.1 (1.1)	11.8 (0.8)	10.7 (0.8)	0.528
density of large intact peltate trichomes (number per mm^2^)				
upper	1.2 (0.1)	1.4 (0.3)	1.1 (0.1)	0.469
lower	3.5 (0.2)ab	4.1 (0.5)a	2.8 (0.3)b	**0.045**

a Different letters indicate statistical difference (*P* < 0.05) between the treatments. *P*-values from ANOVA, *P* < 0.05 **emboldened.** See Figure 1 and Table 1 for details of spectra.

### Phenolics

**Figure 4:**
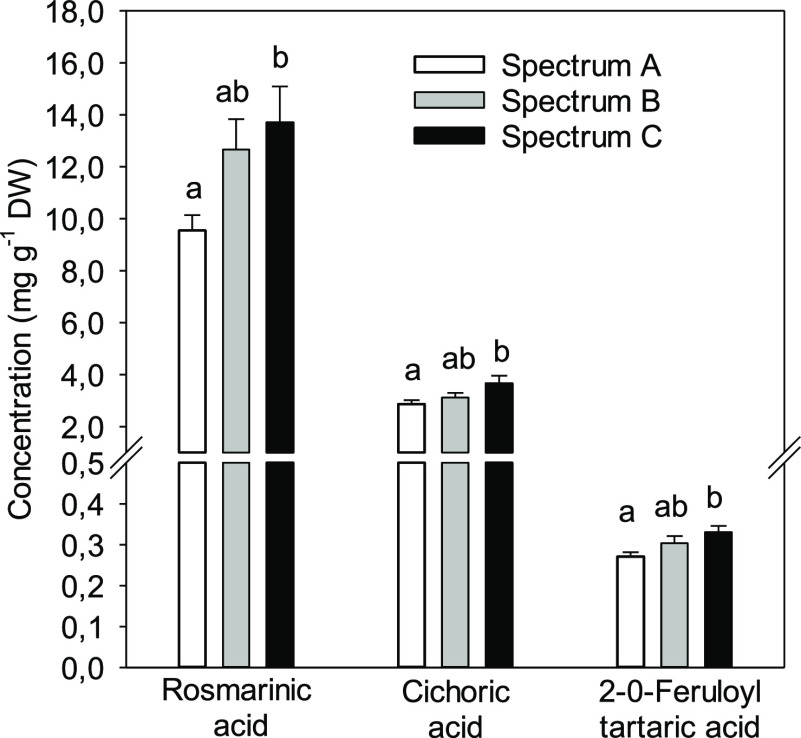
Concentrations of the major phenolic acids in leaves of basil grown under three different LED spectra. Different letters above the bars show a significant difference (*P* < 0.05) between the treatments. Average values with +s.e. are shown (*n* = 11). See Figure 1 and Table 1 for details of spectra.

Concentrations of methanol-extracted phenolics, in general, were the lowest in spectrum A and highest in spectrum C (Figure 4), (Supplementary Table 1). Spectrum C had the highest concentrations of rosmarinic acid, cichoric acid, and 2–0-feruloyl tartaric acid and the difference was significant compared with spectrum A (Figure 4). The concentration of the main compound rosmarinic acid was 8% higher in spectrum C that in than B and 46% higher in C than that in A. Concentrations of chlorogenic acid A, *p*-OH-cinnamic acid derivative, and one lignan-like compound were significantly lower in the spectrum A than those in B (Supplementary Table 1).

### Terpenes

**Figure 5:**
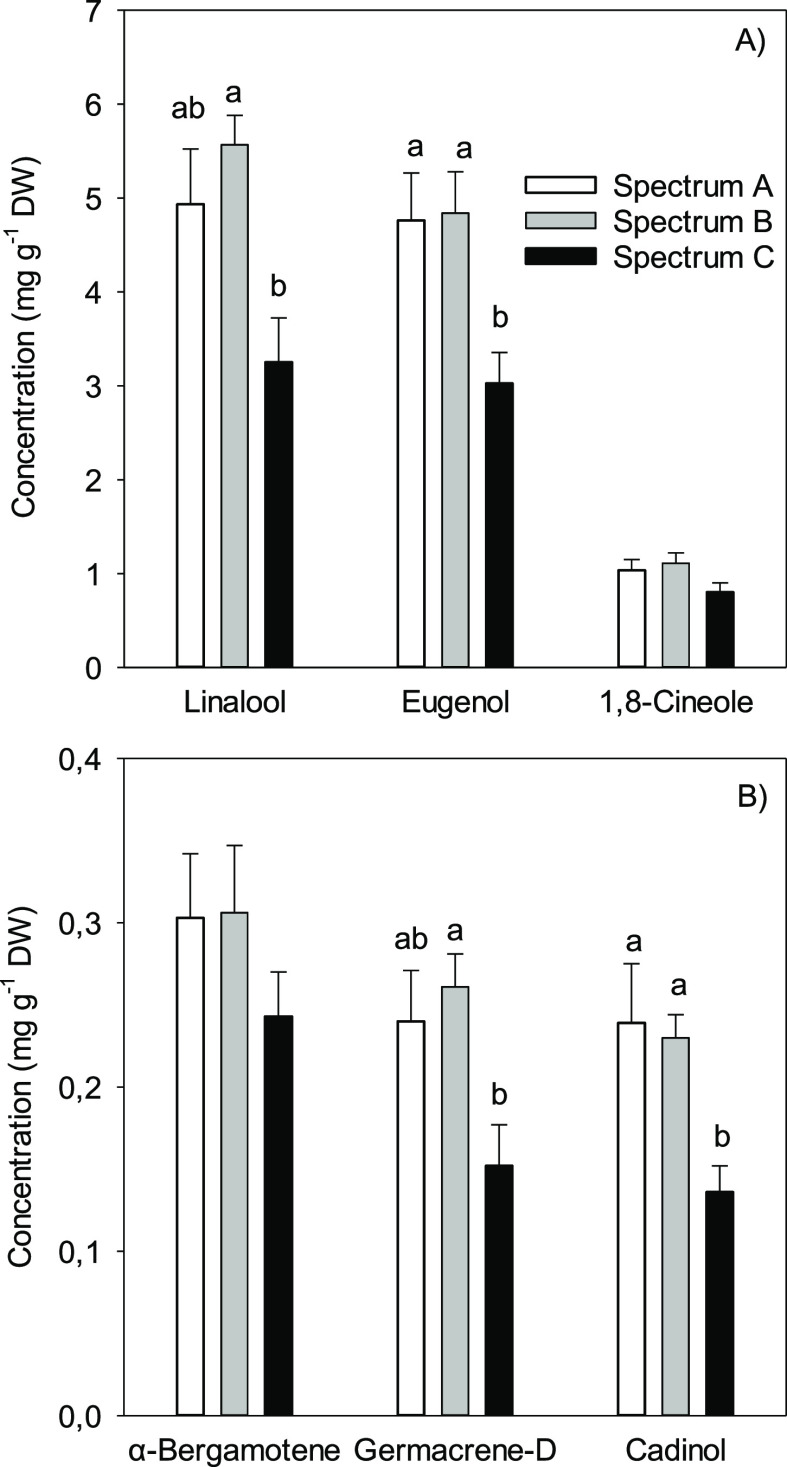
Concentrations of (A) major compounds and (B) major sesquiterpenes in terpene extracts in leaves of basil grown under three different LED spectra. Different letters above the bars show a significant difference (*P* < 0.05) between the treatments. Average values with +s.e. are shown, *n* = 7 for spectrum A, *n* = 6 for spectrum B, and *n* = 8 for spectrum C. See Figure 1 and Table 1 for details of spectra.

Terpene extracts contained 31 terpenoid compounds, phenylpropanoid eugenol, and C6-compound (*E*)-2-hexenal (Supplementary Table 2). A majority of the compounds were oxygenated monoterpenes, linalool (36% of total concentration) and 1,8-cineole (8%) being the main compounds (Figure 5A). Eugenol (Figure 5A) contributed 33% of the total concentration and (*E*)-2-hexenal (Supplementary Table 2) 4%. Sesquiterpenes contributed 13% of the total concentrations and α-bergamotene, germacrene-D, and cadinol had the highest concentrations (Figure 5B). Concentration of 13 compounds altogether as well as total concentrations of sesquiterpenes and oxygenated monoterpenes were affected by the light spectra (Supplementary Table 2).

Concentrations of most compounds were the lowest in spectrum C and differed significantly from that in spectrum B (Figure 5, Supplementary Table 2). The concentration of linalool was 42% lower in spectrum C than that in B and 34% lower in C than that in A. The concentration of eugenol was 60% lower in spectrum C than that in B and 57% lower in C than that in A.

### VOCs

**Figure 6:**
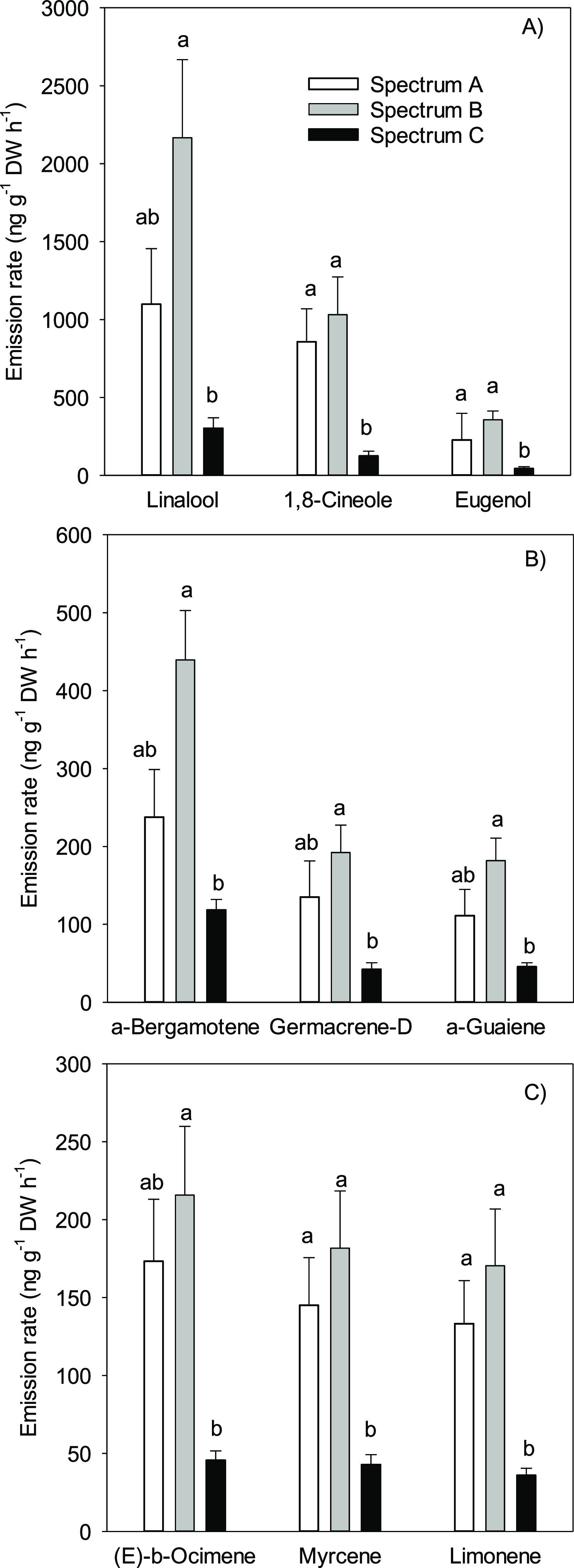
Emission rates of (A) major oxygenated monoterpenes and phenylpropanoid eugenol, (B) major sesquiterpenes, and (C) major nonoxygenated monoterpenes from living basil grown under three different LED spectra. Different letters above the bars show a significant difference (*P* < 0.05) between the treatments. Average values with +s.e. are shown (*n* = 10). See Figure 1 and Table 1 for details of spectra.

Sixty-six different compounds were detected from basil emissions (Supplementary Table 3). When averaged over all the treatments, half of the emissions consisted of oxygenated monoterpenes. Main compounds were monoterpene linalool contributing 30%, monoterpene 1,8-cineole 17%, and phenylpropanoid eugenol 5% (Figure 6A) of the total emissions, respectively. Sesquiterpenes were the second largest compound group, 26% of the total emissions, and the main compounds were α-bergamotene (7% of total emissions), germacrene-D (3%), and α-guaiene (3%) (Figure 6B). Nonoxygenated monoterpenes consisted 19% of the total VOC emissions, and the compounds with largest emissions were (*E*)-β-ocimene, myrcene, and limonene (each contributing 3% of the total emissions) (Figure 6C). GLVs were less than 1% of the emissions. Emission rates of almost all compounds were significantly lower in spectrum C compared with those in spectra A and B (Figure 6, Supplementary Table 3).

In summary, spectrum C, which had the highest share of red and far-red wavelengths but the lowest share of blue and green + yellow wavelengths and the lowest red:far-red ratio (Table 1), was characterized by highest biomass, lowest water content, longest internodes, lowest concentrations of photosynthetic pigments and terpenoids, lowest emissions of VOCs, but highest concentrations of phenolic acids and highest proportion of epidermal cells with phenolics (Table 6). Spectrum A with the highest share of blue and green + yellow wavelengths, the lowest share of red and far-red wavelengths but the highest red:far-red ratio (Table 1) had largest leaves and lowest concentrations of major phenolic compounds (Table 6). Spectrum B which was intermediate between spectra A and C in terms of share of blue, red, far-red, and green + yellow wavelengths (Table 1) had highest concentrations of photosynthetic pigments, smallest leaves, highest density of glandular trichomes, and together with spectrum A, highest concentrations terpenes and BVOC emission rates (Table 6).

**Table 6:**
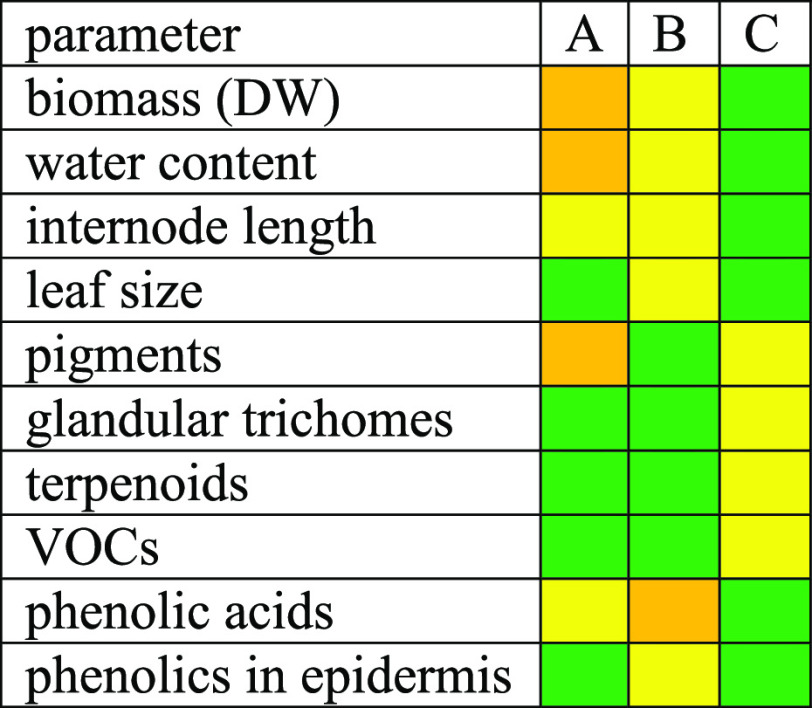
Summary of the Key Results^a^

a Green indicates the highest and yellow the lowest value, orange is the intermediate. See Table 1 for spectrum characteristics and Tables 2–5 and Supplementary Tables 1–3 for the exact parameter values and statistical differences.

## Discussion

Our results showed that light spectra with linearly changing proportions and ratios of UV-A, blue, green+yellow, red, and far-red wavelengths created by LED lights not only affected growth, concentrations of pigments, phenolics, and terpenoids, as well as emissions of volatile compounds of basil leaves but also leaf anatomical parameters related to secondary chemistry. The regulation of biochemical pathways or the role of various photoreceptors was not studied here. Thus, the discussion about the potential mechanisms affecting the observed responses is careful.

In terms of secondary chemistry, spectrum C differed most from the other spectra by having highest concentrations of phenolic acids (rosmarinic acid, cichoric acid, and 2-0-feruloyl tartaric acid) and anthocyanins but lowest concentrations of terpenoids and phenylpropanoid eugenol and their emissions. With the lowest proportion of blue wavelengths and the highest proportion of red wavelengths and increased phenolic acids and anthocyanins in spectrum C, our study supports the previous suggestion^23^ that red light is important in regulating phenolics accumulation in basil. The linear increase of phenolic acids from spectra A to C with linearly decreasing red:far-red ratios are in line with the earlier study reporting linear decrease in concentrations of rosmarinic acid in basil to increasing red:far-red ratios.^24^ Our study gives further support for this study’s conclusion that the phytochrome system is involved in controlling rosmarinic acid accumulation in basil.^24^ Opposite responses in terpenoids and most phenolics here suggest a shift in secondary metabolism routes between terpenoids and phenolic acids by light spectra. Corresponding shifts may have occurred also in the phenylpropanoid biosynthetic pathway, such as *p*-coumaroyl being used more for 4-hydroxy-phenyllactic acid and to further rosmarinic acid synthesis and less for shikimic acid and to further eugenol synthesis in spectrum C.^11^ The shift from terpenoid metabolism to phenolic acids may be interconnected with primary metabolism and growth, because concentrations of photosynthetic pigments chlorophyll a and carotenoids were reduced under spectrum C but growth in terms of DW accumulation increased. However, net photosynthesis was not affected. Potentially, more carbon was allocated to biomass accumulation and phenolic compounds and less to metabolically costly terpenoids and peltate glandular trichomes^39^ in spectrum C. This would also explain lower growth but highest terpenoid concentrations and emission and highest density of glandular trichomes in spectrum B. Eugenol and terpenoids of basil leaves are synthesized and stored in peltate glandular trichomes^9,29^ while phenolic acids are synthetized both in the peltate glandular trichomes and in the leaf tissues.^11^ Changes in concentrations and emissions of eugenol and terpenoids are in line with changes in density of peltate glandular trichomes between the spectra, lowest in C and higher in B and A, but the trend was opposite between concentrations of phenolic acids and peltate glandular trichomes. This suggests that in spectrum C, high share of phenolic acid synthesis took place in leaf tissues other than peltate glandular trichomes. This is partly supported by the increased proportion of cells with phenolics accumulation in the epidermis cells in spectrum C as well as concentrations of anthocyanins that are found in epidermis cells of basil.^40^

In line with spectrum C with the highest proportion of red (600–700 nm) wavelengths and low terpenoid concentrations and emissions, lower essential oil (monoterpenes) contents in basil grown under monochromatic red light (660 nm) compared with monochromatic blue light or white light were earlier reported.^25^ In contrast to our study, increased essential oil contents were reported in species of *Mentha* grown under red LEDs or LEDs with a 70:30 ratio of red:blue compared with field conditions.^41^ The differences may arise from species differences^23^ in responses or the role of proportions or ratios of light wavelengths other than just those of blue and red. The production of volatile compounds in basil was reported to be higher in spectra where green and yellow wavelengths were added to blue and red wavelengths.^15^ In our study, proportion of green and yellow wavelengths was the lowest in spectrum C with lowest emission rates of volatile compounds. Additionally, the lowest red:far-red ratio (similar to shady growing conditions) of spectrum C may be the reason for reduced terpenoid concentrations and emission rates similar to those reported in *Arabidopsis thaliana* and *Hordeum vulgare*.^42,43^ One possible reason for the reduction is the suppression of the signal molecule jasmonic acid controlling terpenoid synthesis.^18^ The low red:far-red ratio also explains the longest internodes and the increased shoot dry weight biomass in spectrum C.^19,44^ Despite the lowest red:far-red ratio in spectrum C, other typical characteristics of shade plants, such as increased petiole and leaf lengths, wider and thinner leaves (due to thinner epidermis and palisade tissues), reduced stomatal density and conductance, increased chlorophyll concentration, reduced net photosynthesis, reduced water loss,^19,45^ or reduced phenolics in basil^40^ were not observed in this study.

Wavelength proportions and their ratios changed linearly from spectrum A to spectrum C; however, the plant responses other than phenolic acid concentrations were not linear. For example, similar to spectrum C, phenolics were accumulated in the upper epidermis in spectrum A, although phenolic concentrations at the whole leaf level were the lowest in spectrum A. The reason may be the highest proportion of UV-A and blue wavelengths in spectrum A, known to induce phenolic accumulation especially in the adaxial (upper) epidermis protecting plants against high-energy radiation.^40,46^ Spectrum B together with spectrum A had the highest terpene concentrations and emission and density of glandular trichomes suggesting a significant role of the emissions from terpene storages. Higher concentrations of chlorogenic acid, which is an intermediate compound in lignin biosynthesis,^47^ as well as one lignan-like compound in spectrum B than A would suggest carbon allocation to cell wall synthesis under these spectral conditions. However, the potential changes in cell wall synthesis were not reflected in growth and DW accumulation which were lower in spectrum B than A suggesting additional roles of chlorogenic acid in spectrum B, such as protection against high-energy radiation.^47^ Increased carotenoids with antioxidative properties against light stress would be in line with this.^48^ Lack of linear responses to increased blue or red wavelengths, e.g., in leaf growth, may also be due to green wavelengths that have both suppressing and enhancing effects on plant performance.^14,49,50^

This study confirms the conclusions of several previous studies that changing light spectra has great potential to modify plant growth and concentrations of secondary chemical compounds that can affect both taste and aroma of basil and also have health-promoting influence. For example, modifying light spectra by LEDs would be an efficient way to increase VOC emissions and enhance plant aroma. The topic needs further studies using different basil cultivars and growing conditions, including shorter cultivation time. The study provides new information showing that while concentration of one chemical group and growth of basil can be increased by a certain light spectrum created by LEDs, the concentrations or volatile emissions of other important compounds decrease. If rosmarinic acid (phenolic acid) and growth in terms of DW are increased in red and far-red dominated spectra, the concentrations and emissions of eugenol and linalool (volatile phenylpropanoid and terpenoid) as well as water content would be decreased. The balance of various chemical compounds and dry weight and water content altered by light spectra may affect taste and texture of basil that should be further studied using sensory evaluation methods.

## Supporting Information Available

Supplementary method description of the chemical fixation method of basil leaves for SEM; concentrations of methanol-extracted phenolics and total phenolics in leaves of basil grown under three different LED spectra (Supplementary Table 1); concentrations of individual and total compounds in terpene extracts in leaves of basil grown under three different LED spectra (Supplementary Table 2); and emission rates of individual and total volatile compounds from living basil plants grown under three different LED spectra (Supplementary Table 3) (PDF)

## References

[ref1] Makri O.; Kintzios S. *Ocimum* sp. (Basil): Botany, Cultivation, Pharmaceutical Properties, and Biotechnology. J. Herbs, Spices Med. Plants 2008, 13, 123–150. 10.1300/J044v13n03_10.

[ref2] Iijima Y.; Davidovich-Rikanati R.; Fridman E.; Gang D. R.; Bar E.; Lewinsohn E.; Pichersky E. The biochemical and molecular basis for the divergent patterns in the biosynthesis of terpenes and phenylpropenes in the peltate glands of three cultivars of basil. Plant Physiol. 2004, 136, 3724–3736. 10.1104/pp.104.051318. 15516500 PMC527170

[ref3] Klimánková E.; Holadová K.; Hajšlová J.; Čajka T.; Poutska J.; Koudela M. Aroma profiles of five basil (*Ocimum basilicum* L.) cultivars grown under conventional and organic conditions. Food Chem. 2008, 107, 464–472. 10.1016/j.foodchem.2007.07.062.

[ref4] Kwee E. M.; Niemeyer E. D. Variations in phenolic composition and antioxidant properties among 15 basil (*Ocimum basilicum* L.) cultivars. Food Chem. 2011, 128, 1044–1050. 10.1016/j.foodchem.2011.04.011.

[ref5] Suppakul P.; Miltz J.; Sonneveld K.; Bigger S. W. Antimicrobial properties of basil and its possible application in food packaging. J. Agric. Food Chem. 2003, 51, 3197–3207. 10.1021/jf021038t. 12744643

[ref6] Lattanzio V.; Lattanzio V. M. T.; Cardinali A. Role of phenolics in the resistance mechanisms of plants against fungal pathogens and insects. In Phytochemistry: Advances in Research, Imperato F., Ed.; Research Signpost: Kerala, 2006; pp 23–67.

[ref7] Loreto F.; Dicke M.; Schnitzler J.-P.; Turlings T. C. J. Plant volatiles and the environment. Plant, Cell Environ. 2014, 37, 1905–1908. 10.1111/pce.12369. 24811745

[ref8] Gonzáles-Burgos E.; Gómez-Serranillos M. P. Terpene compounds in nature: A review of their potential antioxidant activity. Curr. Med. Chem. 2012, 19, 5319–5341. 10.2174/092986712803833335. 22963623

[ref9] Gang D. R.; Wang J.; Dudareva N.; Nam K. H.; Simon J. E.; Lewinsohn E.; Pichersky E. An investigation of the storage and biosynthesis of phenylpropenes in sweet basil. Plant Physiol. 2001, 125, 539–555. 10.1104/pp.125.2.539. 11161012 PMC64856

[ref10] Werker E.; Putievsky E.; Ravid U.; Duda N.; Katzir I. Glandular hairs and essential oil in developing leaves of *Ocimum basilicum* L. *(Lamiaceae)*. Ann. Bot. 1993, 71, 43–50. 10.1006/anbo.1993.1005.

[ref11] Gang D. R.; Beuerle T.; Ullmann P.; Werck-Reichhart D.; Pichersky E. Differential production of *meta* hydroxylated phenylpropanoids in sweet basil peltate glandular trichomes and leaves is controlled by the activities of specific acyltransferases and hydroxylases. Plant Physiol. 2002, 130, 1536–1544. 10.1104/pp.007146. 12428018 PMC166672

[ref12] Olle M.; Viršilė A. The effects of light-emitting diode lighting on greenhouse plant growth and quality. Agric. Food Sci. 2013, 22, 223–234. 10.23986/afsci.7897.

[ref13] Holopainen J. K.; Kivimäenpä M.; Julkunen-Tiitto R. New light for phytochemicals. Trends Biotechnol. 2018, 36, 7–10. 10.1016/j.tibtech.2017.08.009. 28939181

[ref14] Folta K. M.; Maruhnich S. A. Green light: a signal to slow down or stop. J. Exp. Bot. 2007, 58, 3099–3111. 10.1093/jxb/erm130. 17630292

[ref15] Carvalho S. D.; Schwieterman M. L.; Abrahan C. E.; Colquhoun T. A.; Folta K. M. Light quality dependent changes in morphology, antioxidant capacity, and volatile production in sweet basil (*Ocimum basilicum*). Front. Plant Sci. 2016, 7, 1328 10.3389/fpls.2016.01328. 27635127 PMC5007804

[ref16] Han T.; Vaganov V.; Cao S.; Li Q.; Ling L.; Cheng X.; Peng L.; Zhang C.; Yakovlev A. N.; Zhong Y.; Tu M. Improving “color rendering” of LED lighting for the growth of lettuce. Sci. Rep. 2017, 7, 45944 10.1038/srep45944. 28368019 PMC5377472

[ref17] Huché-Thélier L.; Crespel L.; Le Gourrierec J.; Morel P.; Sakr S.; Leduc N. Light signaling and plant responses to blue and UV-radiations – Perspectives for applications in horticulture. Environ. Exp. Bot. 2016, 121, 22–38. 10.1016/j.envexpbot.2015.06.009.

[ref18] Ballaré C. L. Light regulation of plant defense. Annu. Rev. Plant Biol. 2014, 65, 335–363. 10.1146/annurev-arplant-050213-040145. 24471835

[ref19] Demotes-Mainard S.; Péron T.; Corot A.; Bertheloot J.; Le Gourrierec J.; Pelleschi-Travier S.; Crespel L.; Morel P.; Huché-Thélier L.; Boumaza R.; Vian A.; Guérin V.; Leduc N.; Sakr S. Plant responses to red and far-red lights, applications in horticulture. Environ. Exp. Bot. 2016, 121, 4–21. 10.1016/j.envexpbot.2015.05.010.

[ref20] Sipos L.; Balázs L.; Székely G.; Jung A.; Sárosi S.; Radácsi P.; Csambalik L. Optimization of basil (*Ocimum basilicum* L.) production in LED environments – a review. Sci. Hortic. 2021, 289, 110486 10.1016/j.scienta.2021.110486.

[ref21] Taulavuori E.; Taulavuori K.; Holopainen J. K.; Julkunen-Tiitto R.; Acar C.; Dincer I. Targeted use of LEDs in improvement of production of efficiency through phytochemical enrichment. J. Sci. Food Agric. 2017, 97, 5059–5064. 10.1002/jsfa.8492. 28631264

[ref22] Bantis F.; Ouzounis T.; Radoglou K. Artificial LED lighting enhances growth characteristics and total phenolic content of *Ocimum basilicum*, but variably affects transplant success. Sci. Hortic. 2016, 198, 277–283. 10.1016/j.scienta.2015.11.014.

[ref23] Taulavuori K.; Hyöky V.; Oksanen J.; Taulavuori E.; Julkunen-Tiitto R. Species-specific differences in synthesis of flavonoids and phenolic acids under increased periods of enhanced blue light. Environ. Exp. Bot. 2016, 121, 145–150. 10.1016/j.envexpbot.2015.04.002.

[ref24] Schwend T.; Prucker D.; Peisl S.; Nitsopoulos A.; Mempel H. The rosmarinic acid content of basil and borage correlates with the ratio of red and far-red. Eur. J. Hortic. Sci. 2016, 81, 243–247. 10.17660/eJHS.2016/81.5.2.

[ref25] Amaki W.; Yamazaki N.; Ichimura M.; Watanabe H. Effects of light quality on the growth and essential oil content in Sweet basil. Acta Hortic. 2011, 907, 91–94. 10.17660/ActaHortic.2011.907.9.

[ref26] Pennisi G.; Blasioli S.; Cellini A.; Maia L.; Crepaldi A.; Braschi I.; Spinelli F.; Nicola S.; Fernandez J. A.; Stanghellini C.; Marcelis L. F. M.; Orsini F.; Gianquinto G. Unraveling the role of red:blue LED lights on resource use efficiency and nutritional properties of indoor grown sweet basil. Front. Plant Sci. 2019, 10, 305 10.3389/fpls.2019.00305. 30918510 PMC6424884

[ref27] Piesik D.; Łyszczarz A.; Tabaka P.; Lamparski R.; Bocianowski J.; Delaney K. J. Volatile induction of three cereals: influence of mechanical injury and insect herbivory on injured plants and neighbouring uninjured plants. Ann. Appl. Biol. 2010, 157, 425–434. 10.1111/j.1744-7348.2010.00432.x.

[ref28] Kivimäenpä M.; Virjamo V.; Ghimire R. P.; Holopainen J. K.; Julkunen-Tiitto R.; Martz F.; Nissinen K.; Riikonen J. Changes in light spectra modify secondary compound concentrations and BVOC emissions of Norway spruce seedlings. Can. J. For. Res. 2021, 51, 1218–1229. 10.1139/cjfr-2020-0120.

[ref29] Xie Z.; Kapteyn J.; Gang D. R. A systems biology investigation of the MEP/terpenoid and shikimate/phenylpropanoid pathways points to multiple levels of metabolic control in sweet basil glandular trichomes. Plant J. 2008, 54, 349–361. 10.1111/j.1365-313X.2008.03429.x. 18248593

[ref30] Smith H. Light quality, photoperception, and plant strategy. Annu. Rev. Plant Physiol. 1982, 33, 481–518. 10.1146/annurev.pp.33.060182.002405.

[ref31] R Core Team. R: A Language and Environment for Statistical Computing. R Foundation for Statistical Computing, Vienna, Austria. https://www.R-project.org/ (accessed September 6, 2022)

[ref32] Aphalo, P. J. R package for photobiology. (http://www.r4photobiology.info) (accessed September 6, 2022)

[ref33] Kivimäenpä M.; Sutinen S.; Calatayud V.; Sanz M. J. Visible and microscopic needle alterations of mature Aleppo pine (*Pinus halepensis*) trees growing on an ozone gradient in eastern Spain. Tree Physiol. 2010, 30, 541–554. 10.1093/treephys/tpq012. 20215119

[ref34] Lichtenthaler H. K.; Buschmann C. Chlorophylls and Carotenoids: Measurement and Characterization by UV-VIS Spectroscopy. Curr. Protoc. Food Anal. Chem. 2001, F4.3.1–F4.3.8. 10.1002/0471142913.faf0403s01.

[ref35] Männistö E.; Holopainen J. K.; Häikiö E.; Klemola T. A field study with geometrid moths to test the coevolution hypothesis of red autumn colours in deciduous trees. Entomol. Exp. Appl. 2017, 165, 29–37. 10.1111/eea.12626.

[ref36] Taulavuori K.; Julkunen-Tiitto R.; Hyöky V.; Taulavuori E. Blue mood for superfood. Nat. Prod. Commun. 2013, 8, 791–794. 10.1177/1934578X1300800627.

[ref37] Hartikainen K.; Nerg A.-M.; Kivimäenpä M.; Kontunen-Soppela S.; Mäenpä M.; Oksanen E.; Rousi M.; Holopainen T. Emissions of volatile organic compounds and leaf structural characteristics of European aspen (*Populus tremula*) grown under elevated ozone and temperature. Tree Physiol. 2009, 29, 1163–1173. 10.1093/treephys/tpp033. 19448266

[ref38] Mofikoya A. O.; Miura K.; Ghimire R. P.; Blande J. D.; Kivimäenpä M.; Holopainen T.; Holopainen J. K. Understorey *Rhododendron tomentosum* and leaf trichome density affect mountain birch VOC emissions in the Subarctic. Sci. Rep. 2018, 8, 13261 10.1038/s41598-018-31084-3. 30185795 PMC6125604

[ref39] Gershenzon J. Metabolic costs of terpenoid accumulation in higher plants. J. Chem. Ecol. 1994, 20, 1281–1328. 10.1007/BF02059810. 24242341

[ref40] Tattini M.; Landi M.; Brunetti C.; Giordano C.; Remorini D.; Gould K. S.; Guidi L. Epidermal coumaroyl anthocyanins protect sweet basil against excess light stress: multiple consequences of light attenuation. Physiol. Plant. 2014, 152, 585–598. 10.1111/ppl.12201. 24684471

[ref41] Sabzalian M. R.; Heydarizadeh P.; Zahedi M.; Boroomand A.; Agharokh M.; Sahba M. R.; Schoefs B. High performance of vegetables, flowers, and medicinal plants in a red-blue LED incubator for indoor plant production. Agron. Sustainable Dev. 2014, 34, 879–886. 10.1007/s13593-014-0209-6.

[ref42] Kegge W.; Weldegergis B. T.; Soler R.; Vergeer-Van Eijk M.; Dicke M.; Voesenek L. A. C. J.; Pierik R. Canopy light cues affect emission of constitutive and methyl jasmonate-induced volatile organic compounds in *Arabidopsis thaliana*. New Phytol. 2013, 200, 861–874. 10.1111/nph.12407. 23845065 PMC4283982

[ref43] Kegge W.; Ninkovic V.; Glinwood R.; Welschen R. A. M.; Voesenek L. A. C. J.; Pierik R. Red:far-red light conditions affect the emission of volatile organic compounds from barley (*Hordeum vulgare*), leading to altered biomass allocation in neighbouring plants. Ann. Bot. 2015, 115, 961–970. 10.1093/aob/mcv036. 25851141 PMC4407068

[ref44] Xu Y.; Wang C.; Zhang R.; Ma C.; Dong S.; Gong Z. The relationship between internode elongation of soybean stems and spectral distribution of light in the canopy under different plant densities. Plant Prod. Sci. 2021, 24, 326–338. 10.1080/1343943X.2020.1847666.

[ref45] Larcher W. Physiological plant ecology, 3rd ed.; Springer: Berlin, 1995.

[ref46] Siipola S. M.; Kotilainen T.; Sipari N.; Morales L. O.; Lindfors A. V.; Robson T. M.; Aphalo P. J. Epidermal UV-A absorbance and whole-leaf flavonoid composition in pea respond more to solar blue light than to solar UV radiation. Plant, Cell Environ. 2015, 38, 941–952. 10.1111/pce.12403. 25040832

[ref47] Volpi e Silva N.; Mazzafera P.; Cesarino I. Should I stay or should I go: are chlorogenic acids mobilized towards lignin biosynthesis?. Phytochemistry 2019, 116, 112063 10.1016/j.phytochem.2019.112063. 31280091

[ref48] Sandmann G. Antioxidant protection from UV- and light-stress related to carotenoid structures. Antioxidants 2019, 8, 219 10.3390/antiox8070219. PMC6680902 31336715

[ref49] Johkan M.; Shoji K.; Goto F.; Hahida S.; Yoshihara T. Effect of green light wavelength and intensity on photomorphogenesis and photosynthesis in *Lactuca sativa*. Environ. Exp. Bot. 2012, 75, 128–133. 10.1016/j.envexpbot.2011.08.010.

[ref50] Schenkels L.; Sayes W.; Lauwers A.; De Proft M. P. Green light induces shade avoidance to alter plant morphology and increases biomass production in *Ocimum bacilicum* L. Sci. Hortic. 2020, 261, 109002 10.1016/j.scienta.2019.109002.

