# Supplementary material for: Alteration in Light Spectra Causes Opposite Responses in Volatile Phenylpropanoids and Terpenoids Compared with Phenolic Acids in Sweet Basil (Ocimum basilicum) Leaves
Source: J Agric Food Chem. 2022 Sep 20;70(39):12287–96. doi: 10.1021/acs.jafc.2c03309 (PMC9545148; doi:10.1021/acs.jafc.2c03309)
Supplement: Supplementary file 1 — jf2c03309_si_001.pdf [file jf2c03309_si_001.pdf]

Supplementary method description

Chemical fixation of basil leaves for Scanning Electron Microscopy (SEM)

Two leaf segments of ca. 3 x 3 mm next to the midrib of fresh leaves were cut with a razor blade. The segments were kept cold (+4 °C) fixative (2.5 % glutaraldehyde in 0.1 M cacodylate buffer, pH 7.4) overnight. Further steps were done at room temperature. Samples were rinsed with 0.1 M cacodylate buffer for 2 x 10 min, dehydrated in increasing ethanol series, 50 %, 60 %, 70 %, 80 %, 90 % and 94 % for 15 min, and in absolute ethanol 2 x 15 min. Samples were dried in hexamethyldisilazane (HMDS) for 2 x 10 min. Samples were lifted from HMDS on filter paper and let dry. One leaf segment was placed adaxial (upper) leaf side and the other segment abaxial (lower) leaf side upwards on self-adhesive copper tape on aluminum stubs. Samples were sputtered with ca. 50 nm layer of gold (Automatic Sputter Coater B7341, Agar Scientific Ltd., Stansted, UK) and studied by SEM (HR-SEM; Carl Zeiss, Sigma HD|VP, Oberkochen, Germany).

Supplementary Table 1. Average (s.e., *n*=11) concentrations (mg/g DW) of methanol-extracted phenolics with absorbances (nm) used for quantification in leaves of basil grown under three different LED spectra. *P*-values from ANOVA^{A}, Welch^{W} or Kruskal-Wallis test* are shown, *P*<0.05 **emboldened.** Different letters indicate significant difference (*P*<0.005) between the treatments.

	RT (min)	Spectrum A	Spectrum B	Spectrum C	*P*
Lignan 1[1] (A220)	2.166	0.104 (0.010)	0.121 (0.025)	0.127 (0.021)	0.708^{A}
Lignan 2[1] (A220)	2.375	0.153 a (0.021)	0.288 b (0.035)	0.203 ab (0.040)	**0.026**$^{K\text{-}W}$
Gallotannin[2] (A 220)	7.761	0.009 (0.001)	0.012 (0.001)	0.011 (0.001)	0.079^{A}
Chlorogenic acid (A 320)	9.809	0.343 a (0.011)	0.454 b (0.030)	0.366 ab (0.388)	**0.006**^{A}
Chlorogenic acid der (A 320)	10.288	0.079 (0.010)	0.088 (0.013)	0.060 (0.010)	0.179^{A}
p-OH-cinnamic acid der (A320)	11.627	0.056 a (0.002)	0.071 b (0.003)	0.063 ab (0.005)	**0.002**^{W}
Cichoric acid (A320)	20.968	2.870 a (0.143)	3.120 ab (0.179)	3.655 b (0.301)	**0.048**^{A}
2-0-Feruloyl tartaric acid (A320)	23.331	0.271 a (0.011)	0.304 ab (0.017)	0.330 b (0.016)	**0.033**^{A}
Rosmarinic acid (A320)	24.017	9.550 a (0.588)	12.664 ab (1.171)	13.695 b (1.402)	**0.034**^{A}
Lignan 3[1] (A220)	24.774	0.466 (0.057)	0.562 (0.087)	0.589 (0.124)	0.754$^{K\text{-}W}$
Total		13.902 a (0.691)	17.684 ab (1.360)	19.099 b (1.778)	**0.013**^{W}

[1]identified based on their similar UV-spectrum with lignans, [2]tentatively identified as trigalloylglucose. RT = retention time.

Supplementary Table 2. Average (s.e.) compound concentration (mg/g DW) in terpene extracts in leaves of basil grown under three different LED spectra. *P*-values of ANOVA, Welch[W] or Kruskal-Wallis test* are shown, *P*<0.05 **emboldened.** Different letters indicate significant difference (*P*<0.005) between the treatments.

Compound	RT (min)	A (n=7)	B (n=6)	C (n=8)	*P*-value
α-Pinene	7.184	0.034 (0.004)	0.041 (0.003)	0.028 (0.003)	0.052
Camphene	7.555	0.004 (0.002)	0.004 (0.002)	0.001 (0.001)	0.321*
Sabinene	8.166	0.041 (0.005)	0.046 (0.004)	0.031 (0.004)	0.078
β-Pinene	8.257	0.102 (0.010) ab	0.110 (0.009) a	0.075 (0.009) b	**0.037**
Myrcene	8.591	0.108 (0.012)	0.118 (0.015)	0.089 (0.015)	0.369
Limonene	9.623	0.042 (0.008)	0.055 (0.004)	0.044 (0.004)	0.192*
(*Z*)-Ocimene	9.847	0.013 (0.006)	0 (0)	0.007 (0.007)	0.178*
(*E*)-β-Ocimene	10.135	0.227 (0.020)	0.222 (0.024)	0.193 (0.023)	0.504
Terpinolene	11.280	0.014 (0.003)	0.011 (0.004)	0.011 (0.003)	0.706*
**Total non-oxygenated monoterpenes**		0.585 (0.047)	0.606 (0.062)	0.478(0.061)	0.251
1,8-Cineole	9.690	1.035 (0.117)	1.111 (0.112)	0.803 (0.100)	0.137
(*E*)-Sabinene hydrate	10.678	0.031 (0.002)	0.021 (0.009)	0.018 (0.006)	0.244*
Linalool	11.561	4.934 (0.588) ab	5.567 (0.312) a	3.250 (0.474) b	**0.009**
Camphor	12.883	0.085 (0.005)	0.078 (0.008)	0.068 (0.007)	0.191
α-Terpineol	14.177	0.080 (0.008)	0.080 (0.007)	0.057 (0.007)	0.066
Bornyl acetate	16.494	0.014 (0.002)	0.010 (0.003)	0.009 (0.002)	0.352*
Eugenol[a]	17.673	4.761 (0.506) a	4.840 (0.441) a	3.027 (0.329) b	**0.009**
**Total oxygenated monoterpenes**		10.942 (1.165) a	11.704 (0.768) a	7.232 (0.858) b	**0.008**
Unknown sqt1	17.402	0.027 (0.003)	0.025 (0.005)	0.017 (0.003)	0.167
α-Copaene	17.999	0.060 (0.016) ab	0.093 (0.006) a	0.036 (0.014) b	**0.017***
β-Elemene	18.208	0.194 (0.019)	0.222 (0.016)	0.148 (0.022)	0.673
Unknown sqt2	18.588	0.061 (0.006) ab	0.067 (0.003) a	0.042 (0.007) b	**0.012***
α-Bergamotene	18.752	0.303 (0.039)	0.306 (0.041)	0.243 (0.027)	0.938
α-Guaiene	18.807	0.076 (0.008) ab	0.086 (0.004) a	0.055 (0.010) b	**0.043**
Unknown sqt3	18.913	0.027 (0.003) ab	0.029 (0.002) a	0.016 (0.004) b	**0.019***
(*E*)-β-Farnesene	18.958	0.090 (0.011)	0.096 (0.018)	0.079 (0.017)	0.745
α-Humulene	19.023	0.078 (0.007) ab	0.087 (0.006) a	0.064 (0.004) b	**0.034**
Unknown sqt4	19.127	0.047 (0.005)	0.050 (0.004)	0.034 (0.006)	0.081
Germacrene-D	19.335	0.240 (0.031) ab	0.261 (0.020) a	0.152 (0.025) b	**0.019**
Bicyclogermacrene	19.504	0.077 (0.010)	0.081 (0.005)	0.055 (0.008)	0.060
δ-Guaiene	19.587	0.100 (0.012)	0.112 (0.006)	0.073 (0.011)	0.053
γ-Cadinene	19.677	0.081 (0.010) ab	0.086 (0.005) a	0.057 (0.006) b	**0.029**
δ-Cadinene	19.749	0 (0) a	0.028 (0.003) b	0.014 (0.005) ab	**0.005***
Cadinol	20.902	0.239 (0.036) a	0.230 (0.014) a	0.136 (0.016) b	**0.003[W]**
**Total sesquiterpenes**		1.701 (0.197) ab	1.861 (0.131) a	1.222 (0.157) b	**0.035**
(*E*)-β-Hexenal[b]	5.373	0.341 (0.051) a	0.599 (0.028) b	0.384 (0.033) ac	**<0.001**

[a]phenylpropanoid, included in total oxygenated monoterpenes, [b] six-carbon aldehyde, RT = retention time, sqt = sesquiterpene

Supporting Table 3. Average (s.e., *n* = 10) VOC emission rates (ng g^{-1} DW h^{-1}) from living basil plants grown under three different LED spectra. *P*-values from Kruskal-Wallis test are shown, *P*<0.05 **emboldened**. Different letters indicate statistical difference between the treatments.

Compound	RT (min)	Spectrum A	Spectrum B	Spectrum C	*P*
E)-β-Hexenal	13.19	1.5 (1.5)	0 (0)	0 (0)	0.387
(*Z*)-3-Hexenol	13.25	6.2 (2.7)	14.7 (12.5)	0.9 (0.9)	0.309
1-Octen-3-ol	17.66	12.8 (5.8) ab	30.4 (10.5) a	1.3 (0.9) b	**0.007**
**Total GLVs**		20.6 (6.7) ab	45.0 (22.6) a	2.2 (1.2) b	**0.009**
α-Thujene	15.98	5.3 (1.2) ab	6.5 (1.2) a	1.8 (0.2) b	**0.005**
α-Pinene	16.32	89.6 (18.7) a	109.3 (20.4) a	28.7 (3.0) b	**0.004**
α-Fenchene	16.84	0.7 (0.3)	0.7 (0.3)	0.1 (0.1)	0.135
Camphene	16.93	25.3 (5.3) a	30.4 (7.1) a	6.8 (0.9) b	**0.005**
Verbenene	17.09	1.4 (0.5)	2.0 (0.6)	0.4 (0.2)	0.103
Sabinene	17.74	109.0 (25.2) a	128.0 (22.4) a	34.7 (3.4) b	**0.004**
β-Pinene	17.97	119.9 (25.7) a	147.7 (27.6) a	36.6 (4.1) b	**0.005**
Myrcene	18.16	145.0 (30.6) a	181.7 (36.7) a	42.9 (6.3) b	**0.007**
α-Phellandrene	18.86	6.9 (1.8) ab	9.5 (1.9) a	2.1 (0.5) b	**0.006**
Δ-3-Carene	19.095	1.3 (0.5) ab	2.6 (0.7) a	0.4 (0.2) b	**0.032**
α-Terpinene	19.28	5.0 (1.5) ab	8.0 (1.7) a	2.4 (0.3) b	**0.007**
Cymene	19.56	6.7 (1.9) ab	9.7 (1.9) a	2.6 (0.6) b	**0.009**
Limonene	19.77	133.2 (27.7) a	170.4 (36.4) a	36.0 (4.5) b	**0.004**
β-Phellandrene	19.81	1.3 (1.3) a	6.6 (3.0) ab	8.0 (0.8) b	**0.012**
(*E*)-β-Ocimene	20.21	173.4 (39.7) ab	215.7 (44.1) a	45.7 (6.0) b	**0.005**
γ-Terpinene	20.76	6.8 (1.5) ab	9.2 (1.7) a	3.2 (0.3) b	**0.006**
α-Terpinolene	21.85	43.1 (8.7) a	52.9 (10.9) a	14.0 (1.3) b	**0.005**
allo-Ocimene	23.05	0.7 (0.7)	0.5 (0.5)	0 (0)	0.626
p-Mentha-triene	23.185	2.7 (0.9)	2.0 (0.9)	0.3 (0.2)	0.127
4,8-Dimethyl-1,3,7-nonatriene	22.56	0.7 (0.5)	0.7 (0.5)	7.5 (4.7)	0.081
**Total non-oxygenated mono- and homo-terpenes**		877.3 (187.7) a	1093.5 (211.2) a	274.2 (31.7) b	**0.005**
2,3-Dehydro-1,8-cineole	18.35	1.8 (1.2)	1.4 (1.4)	0.7 (0.5)	0.838
1,8-Cineole	19.96	858.3 (210.9) a	1031.8 (241.6) a	125.4 (30.7) b	**0.004**
(*E*)-Sabinene-hydrate	21.11	2.8 (1.5) ab	4.0 (0.9) a	0.2 (0.2) b	**0.009**
Linalool	22.06	1098.7 (356.1) ab	2166.6 (500.2) a	301.8 (68.1) b	**0.002**
Camphor	24.05	22.0 (10.6) ab	27.4 (6.9) a	3.0 (1.3) b	**0.005**
Menthone	24.20	0.8 (0.3)	1.3 (0.4)	0.2 (0.1)	0.218
Borneol	24.70	3.0 (1.5)	3.4 (1.0)	0.3 (0.3)	0.071
α-Terpineol	25.39	18.9 (7.2) ab	26.5 (3.9) a	5.5 (0.7) b	**0.001**
Bornyl acetate	24.86	9.9 (4.5) ab	13.1 (3.0) a	2.9 (0.4) b	**0.006**
α-Terpineyl acetate	30.26	1.1 (0.5)	1.5 (0.5)	<1 (<1)	0.068
Eugenol[a]	30.67	227.6 (171.4) a	356.7 (57.0)a	44.7 (11.4) b	**0.002**
**Total oxygenated monoterpenes**		2244.0 (733.4) ab	3633.0 (771.9) a	477.3 (102.5) b	**0.002**

Supporting Table 3 continues

Compound	RT (min)	Spectrum A	Spectrum B	Spectrum C	*P*
Unknown sqt1	30.26	0 (0) a	2.5 (1.1) b	0.2 (0.2) ab	**0.044**
Unknown sqt2	30.48	5.5 (2.3)	4.7 (3.5)	6.4 (1.4)	0.319
α-Copaene	31.41	35.1 (8.9) ab	57.0 (9.1) a	17.0 (2.0) b	**0.003**
Unknown sqt3	31.55	3.0 (2.5)	4.9 (2.3)	0.5 (0.5)	0.314
β-Elemene	31.75	53.3 (18.0) ab	74.9 (12.2) a	18.8 (2.8) b	**0.002**
Unknown sqt4	31.92	6.5 (1.7)	8.5 (1.8)	3.1 (0.6)	0.053
(*E*)-α-Bergamotene	32.33	3.1 (1.5)	5.5 (1.6)	0.2 (0.2)	0.061
Unknown sqt5	32.51	0 (0)	1.3 (1.0)	0 (0)	0.140
Unknownt sqt6	32.74	12.1 (3.3) ab	18.5 (2.9) a	4.9 (1.0) b	**0.003**
(*E*)-β-Caryophyllene	32.83	0 (0)	2.0 (2.0)	0 (0)	0.387
α-Bergamotene	32.92	237.6 (61.2) ab	439.3 (63.5) a	118.4 (13.6) b	**0.005**
Unknownt sqt7	33.04	9.9 (2.5) ab	16.8 (2.3) a	5.3 (0.9) b	**0.003**
α-Guiaene	33.15	111.1 (33.7) ab	182.0 (28.9) a	45.7 (4.9) b	**0.002**
Aromadendrene	33.34	7.0 (2.2)	13.1 (3.3)	3.0 (1.1)	0.080
Unknown sqt8	33.46	12.5 (3.4) ab	19.4 (2.9) a	8.5 (0.8) b	**0.019**
Unknown sqt9	33.63	7.0 (2.0) ab	11.2 (1.9) a	2.9 (0.8) b	**0.007**
α-Humulene	33.81	33.0 (8.5) ab	54.3 (9.0) a	14.2 (1.7) b	**0.003**
Unknown sqt10	34.01	29.4 (9.1) ab	48.4 (7.3) a	15.3 (1.8) b	**0.006**
Unknown sqt11	34.23	17.8 (4.8) ab	28.9 (4.3) a	9.9 (1.1) b	**0.004**
Unknown sqt12	34.34	5.2 (3.7) a	30.5 (7.3) b	7.5 (1.6) ab	**0.010**
Germacrene-D	34.52	135.0 (46.5) ab	192.4 (35.0) a	42.1 (8.5) b	**0.002**
Unknown sqt13	34.73	19.2 (5.1) ab	31.3 (4.8) a	12.1 (1.3) b	**0.009**
Unknown sqt14	34.83	5.1 (3.9)	0 (0)	0 (0)	0.140
Unknown sqt15	34.93	60.7 (17.2) ab	99.9 (16.2) a	31.9 (3.8) b	**0.006**
δ-Guaiene	35.08	70.4 (23.6) ab	97.7 (16.6) a	31.4 (4.2) b	**0.004**
δ-Cadinene	35.30	58.4 (20.9) ab	98.1 (15.3) a	30.6 (3.5) b	**0.014**
γ-Cadinene	35.42	16.6 (4.1) ab	23.8 (3.6) a	9.3 (1.0) b	**0.008**
1S-cis-Calamenene	35.50	11.0 (3.1) ab	17.3 (2.6) a	6.2 (0.6) b	**0.005**
Cadina-1,4-diene	35.73	4.3 (1.2) ab	7.2 (1.1) a	3.1 (0.4) b	**0.013**
Unknown sqt16	35.84	5.0 (1.5) ab	8.2 (1.1) a	3.1 (0.4) b	**0.005**
Unknown sqt17	35.96	0 (0)	3.5 (2.3)	0 (0)	0.140
Cadinol	38.07	27.7 (9.4) ab	35.1 (3.5) a	16.5 (1.3) b	**0.012**
**Total sesquiterpenes**		1002.2 (287.1) ab	1637.9 (247.6) a	468.2 (51.6) b	**0.003**

[a]phenylpropanoid, included in total oxygenated monoterpenes, RT = retention time, sqt = sesquiterpene
